# Multidimensional Self-Concept Depending on Levels of Resilience and the Motivational Climate Directed towards Sport in Schoolchildren

**DOI:** 10.3390/ijerph17020534

**Published:** 2020-01-15

**Authors:** Irwin Andrés Ramirez-Granizo, María Sánchez-Zafra, Félix Zurita-Ortega, Pilar Puertas-Molero, Gabriel González-Valero, Jose Luis Ubago-Jiménez

**Affiliations:** 1 Department of Didactics of Musical, Plastic and Corporal Expression, University of Granada, 18071 Granada, Spain; irwinrg@ugr.es (I.A.R.-G.); felixzo@ugr.es (F.Z.-O.); pilarpuertasmolero@gmail.com (P.P.-M.); ggvalero@ugr.es (G.G.-V.); jlubago@ugr.es(J.L.U.-J.); 2 Department of Didactics of Musical, Plastic and Corporal Expression, University of Jaén, 23071 Jaén, Spain

**Keywords:** goal orientation, resilience, self-concept, physical activity, children

## Abstract

(1) *Background*: Motivation towards sports practice is fundamental at an early age, as this can favor the integral development of the student body. (2) *Methods*: The main objective of this study was to describe and analyze the relationships between the different dimensions of self-concept based on motivational climate, body mass index and resilience in a sample of 203 children from the third cycle of primary education, with an age between 11 and 13 years (M = 11.54). They completed the motivational climate questionnaires (PMCSQ-2), the self-concept questionnaire (AF-5) and the questionnaire that measures resilience levels (the Connor–Davidson Resilience Scale (CD-RISC)). (3) *Results*: The results showed that boys are more resilient than girls and, in turn, have a greater tendency to task climate compared to them. Regarding self-concept, males presented higher scores in the academic, social and physical dimensions. In the same line as resilience, the motivational climate in males is oriented to the ego climate and the feminine to the task climate. Negative correlations of physical self-concept were found with the ego and task climate. (4) *Conclusions*: The task climate was identified as a predictor of resilience levels.

## 1. Introduction

Adolescence is a stage of great importance in the creation of healthy habits, since it represents the period of transition to adult life in which lifestyles are configured [1]. During this period, an attempt is made to promote healthy behaviors during childhood, since it is mainly characterized by being a fundamental period in the acquisition and consolidation of active and healthy lifestyles [2,3]. Despite the fact that the child population is the most active age group in the population, levels of physical exercise have been rapidly decreasing [4], which may be related to a high degree of sedentariness, obesity and therefore a high body mass index for their age or stage. Actually, being the most energetic age group does not imply that the practice of physical activity is sufficient to maintain or improve their state of health. Likewise, another of the benefits produced by an active lifestyle are those given at a physiological, psychological or social level, including the development of cardiovascular capacity, providing mental well-being and even improving socio-affective relationships [5,6,7]. Various studies such as those in [8,9] have demonstrated the positive effects of the habit of practicing physical activity, both physiologically and socially, cognitively and affectively. Daily physical activity prevents numerous pathologies such as obesity, thus reducing the body mass index, hypertension, diabetes or certain types of cancer [9]. In addition to allowing the configuration of more affective social relationships [10,11], it helps deal with stressful situations, including depression, and improves self-esteem and self-concept. Estimating the cognitive factors involved in adolescence, along with the importance of following a healthy lifestyle, it is necessary to study some of the cognitive processes that develop at this stage. Self-concept is defined by current psychology as a mental image, composed of different factors, of what a subject or individual thinks of himself [12]. According to studies such as that of García and Musitu [13], this factor comprises five dimensions—social, emotional, physical, academic and family—trying to provide a closer vision of all daily realities.

Given the benefits of physical sports practice, as well as its advantages as a playful and social medium, it is interesting to determine the motivational aspects that operate in the practice of sports in schoolchildren, since many other habits that will be repeated throughout adult life will depend on these [14,15,16]. There are several studies on motivation that qualify it as a substantial element in any type of learning [17,18]. According to this aspect, a motivated student will achieve more significant learning in the educational context, since his own goals will be the objectives to achieve [19]. This is where the role of the teacher appears, trying to involve the students in physical exercise through physical education [17], since the motivation shown by the students will be pivotal in current involvement as well as in future habits [20]. Likewise, the teacher will be pivotal in the creation of an optimal motivational climate that is created in the classroom through an environment of autonomy, playful dynamics and teamwork [21,22,23]. In this way, the motivational climate can be defined as the environment constituted by explicit and implicit signals in an environment, which allow us to constitute patterns of success [24,25,26]. One of the most used theories to explain the motivation towards sport has been the theory of achievement goals [27,28]. This model studies the abilities that each subject possesses, since it determines that the objectives it establishes depend on the perception that the individual has of his or her sports skills [29]. In this way, a person’s motivational climate towards sport can be oriented towards the task climate and the ego climate [30,31,32]. On the one hand, the task climate responds to the good attitude of the student toward the subject and the eagerness to perform challenging tasks, through personal improvement and cooperation, recognizing the effort in the results [21,33], creating positive and favorable attitudes toward exercise. On the contrary, the activities oriented to the ego climate seek the scope of achievements without any effort, marked by a low motivation and interest in the physical education sessions [34], and this dimension is based on social comparison and the search for external recognition.

This topic also includes a new concept in the field of sports psychology, resilience considered as the set of intrinsic qualities that make up an individual’s ability to overcome situations of adversity and provoke stress [35]. Authors such as Wilks and Spivey [36] or Zurita [37] address this issue in relation to the overcoming of stages linked to academic, social or health and mental physics. In fact, one of the most interesting areas deals with resilience and its relationship with sports physical practice, highlighting how sports practice improves the ability to solve problems as well as developing the ability to self-regulate purposes and plans for the future. On the other hand, the practice of physical sports activity allows the intrapersonal knowledge of the subject, thus knowing his weak and strong points and improving self-esteem, levels of self-concept and physical and mental health [38,39], which is why this competition can increase thanks to sport.

Several research projects attempt to answer this question, especially in primary, High School and even university contexts. Among them are those carried out by Ramírez-Granizo [40] in schoolchildren, another study carried out by Sánchez-Zafra [41] as an analysis of violent behavior according to gender and type of center in primary schools [42] or even addressing variables such as violent behavior in school [41] or academic success based on the motivational climate measured by the motivation of university students [43]. The three terms dealt with in this document run along a common axis, which is the important psychosocial component it entails, and also how this model influences gender, since some authors point out a greater involvement towards sports activity if the psychosocial characteristics (self-concept or resilience) are addressed, thus producing improvement at a general level in the quality of life of the subject in question [37,40,41,42].

Based on the foregoing, the main objectives of this study are to describe and analyze the relationships between the motivational climate directed towards sport, the levels of self-concept, body mass index “BMI” and resilience in a sample of schoolchildren in the fifth and sixth grade of primary education in the province of Granada (Spain).

## 2. Materials and Methods

### 2.1. Design and Participants

A cross-sectional and non-experimental study was carried out using a single measurement within a single group. The sample consisted of a total of 203 schoolchildren who attended the fifth and sixth year of primary education in the city of Granada, with a homogeneous distribution according to sex, representing 42.9% (n = 87) for males and 57.1% (n = 116) for females. Convenience sampling was used to recruit the participants, with only school children in the third stage of their primary education who belonged to 6 public and 2 subsidized educational centers in the province being invited to participate. The age of the participants was between 10 and 13 years of age (M = 11.54 years; DT = 0.53).

### 2.2. Instruments

Self-concept. Form-5 Autoconcept Questionnaire (AF-5). This instrument was elaborated by García and Musitu [13] and is based on the theoretical model of Shavelson [44]. It consists of 30 items that are scored using a Likert scale of 5 options, where 1 is “Never” and 5 is “Always”. The self-concept is grouped into five dimensions according to this instrument: academic self-concept (items 1, 6, 11, 16, 21 and 26), social self-concept (items 2, 7, 12, 17, 22 and 27), emotional self-concept (items 3, 8, 13, 18, 23 and 28), family self-concept (items 4, 9, 14, 19, 24 and 29) and physical self-concept (items 5, 10, 15, 20, 25 and 30). The Garcia and Musitu study [13] established a reliability (determined by the Cronbach alpha coefficient) of α = 0.810, a value similar to that detected in this research work at a general level (α = 0.864).

Resilience. The Connor–Davidson Resilience Scale (CD-RISC) [45] was used to determine the resilient scores of each child. It consists of 25 items; the respondent must indicate to what extent each statement has been true in the past month. A Likert scale is used, which is graded with a range of 4 response options from 0 to 4, where 0 represents “Not true at all” and 4 is “True almost always”. The higher the value, the higher the level of resilience. The construct is composed of five factors: persistence-tenacity-self-efficacy (items: 10–12, 16, 17, 23–25); control under pressure (6, 7, 14, 15, 18, 19, 20); adaptability and support networks (1, 2, 4, 5, 8); control and purpose (13, 21, 22); and spirituality (3, 9). Examination of internal consistency produced an acceptable value for Cronbach’s alpha (α = 0.86) at a general level.

Motivational climate. Questionnaire of motivational climate perceived in sport (PMCSQ-2). This questionnaire was originally developed by Newton, Duda and Yin [46] and was adapted to Spanish by González-Cutre, Sicilia and Moreno [43]. It is composed of 33 items, which are scored on a Likert scale of five options, where 1 is “totally disagree” and 5 “totally agree”. This variable is categorized in two dimensions and these are further divided into three subcategories. The task climate “TC” comprises cooperative learning “CL” (items 11, 21, 31 and 33), effort/improvement “EI” (items 1, 8, 14, 16, 20, 25, 28 and 30) and important role “IR” (items 4, 5, 10, 19 and 32). Ego climate “EC” comprises punishment for errors “PE” (items 2, 7, 9, 15, 18 and 27), unequal recognition “UR” (items 3, 13, 17, 22, 24, 26 and 29) and member rivalry “MR” (items 6, 12 and 23). Examination of internal consistency produced an acceptable value for Cronbach’s alpha (α = 0.82) at a general level.

Body mass index (BMI) was calculated from the participant’s weight (in kilograms) divided by the square of their height (in meters). The weight of the participants was measured using a Tanita for the weight and a stadiometer for the height.

Sociodemographic aspects. An ad hoc questionnaire was elaborated for the recording of sociodemographic variables (sex, age, residence, etc.).

### 2.3. Procedure

In the first place, the collaboration of the participants was requested through an informative letter elaborated from the Corporal Area of the University of Granada and was delivered to the school centers. An appointment was also made with the director of the centers, who were given a copy of the questionnaire and an information letter. This detailed the nature and objectives of the study to be carried out, as well as requesting their informed consent to participate in this research. 

Once the proposal was accepted, specific timetables were established for carrying out these questionnaires (physical education class). Data were collected during school hours. The process took place without any incidence, always in the presence of the researchers for correct application of the questionnaire and the resolution of doubts. Once the investigation was finished, a report with the results was delivered to the centers. Anonymity was assured to all students who participated voluntarily, in compliance with the Helsinki Research Ethics Agreement. The Research Ethics Committee of the University of Granada approved this study and a total of 38 questionnaires were invalidated and found to be poorly completed.

### 2.4. Data Analysis

Statistical analysis was performed using IBM SPSS^®^ 22.0 software (IBM, Armonk, NY, USA). For the basic descriptives, frequencies and means were used. For the study of relations between variables, Student’s *T* test, ANOVA of a factor and Pearson’s bivariate correlations were used, depending on the nature of the variables. Data normality was performed using the Kolmogorov–Smirnov test, using Lilliefors correction and homoscedasticity using the Levene test. The internal reliability of the instruments used was evaluated using Cronbach’s alpha coefficient, setting the Reliability Index at 95.5%.

## 3. Results

Table 1 shows the levels of motivational climate, self-concept and resilience of schoolchildren in the fifth and sixth grade, respectively, of primary education in Andalusia. In relation to motivational climate, statistically significant differences were found at level (*p* < 0.05) in the dimensions PE (*p* = 0.04) and MR (*p* = 0.05). It is also observed that the task climate presents similar scores between men and women (4.00 ± 0.52 vs. 3.99 ± 0.54). In males the ego climate was generally higher (2.50 ± 0.80 vs. 2.36 ± 0.80) as well as in the effort-improvement dimension (3.89 ± 0.47 vs. 3.82 ± 0.82). In women, the cooperative learning dimensions (3.99 ± 0.72 vs. 4.01 ± 0.76) and important role (4.11 ± 0.78 vs. 4.13 ± 0.74) had higher mean scores. Based on self-concept levels, statistically significant differences were found at level (*p* < 0.05) in family self-concept (*p* = 0.039) and (*p* < 0.01) in academic category (*p* = 0.005). On the other hand, it is shown that general self-concept is higher in males (2.78 ± 0.20 vs. 2.76 ± 0.21), including for its academic dimensions (2.47 ± 0.48 vs. 2.76 ± 0.21), social (2.91 ± 0.34 vs. 2.82 ± 0.35) and physical (3.91 ± 0.51 vs. 2.35 ± 0.53), while family self-concept was higher in women (2.91 ± 0.33 vs. 2.99 ± 0.23).

Table 2 shows the levels of resilience according to the gender of the participants. The results are homogeneous, since no statistically significant differences were found, but it can be observed that the male sex presented higher scores in all its dimensions, with the exception of optimism and adaptation to stressful situations (3.16 ± 0.68 vs. 3.17 ± 0.69), where the female sex obtains higher values. The highest score was obtained in the control locus (3.22 ± 0.59).

Finally, bivariate correlations are made between the different dimensions of self-concept, resilience, motivational climate and BMI (Table 3). Considering the associations given with academic self-concept, statistically significant differences are obtained with physical self-concept (*p* < 0.01), task climate (*p* < 0.01), adaptability (*p* < 0.01), spirituality (*p* < 0.01), social self-concept (*p* < 0.05) and persistence (*p* < 0.05). All correlations are negative and indirect, except for social self-concept and physical self-concept, with the strongest correlation for adaptability observed (*r* = 0.317). The lowest correlation strength was found with social self-concept (*r* = 0.158). For the social dimension, statistically significant differences were obtained with the family member and the body mass index (*p* < 0.05), the persistence (*p* < 0.01) and control and purpose (*p* < 0.01). It is observed that the positive and direct correlations were with the family dimension and the body mass index, with the strongest correlation with the persistence being observed (*r* = 0.204). The lowest correlation strength was found with the physical self-concept. In the family category, statistically significant differences were obtained with optimism (*p* < 0.01). Correlations are negative and indirect, with the strongest correlation with the OPT dimension (*r* = 0.226) and the weakest in the ego climate (*r* = 0.006). In relation to the physical self-concept, statistically significant differences are obtained with TC, persistence, adaptability and optimism (*p* < 0.01), climate ego (*p* < 0.05). All correlations are negative and indirect, with the strongest correlation with the persistence being observed (*r* = 0.312). The lowest correlation strength was found with ego climate (*r* = 0.149).

On the other hand, if the associations given with the task climate are analyzed, statistically significant differences are obtained with CE, persistence, control under pressure, adaptability and optimism (*p* < 0.01), spirituality (*p* < 0.05). All correlations are positive and direct, except that of the EC, with the strongest correlation with ego climate being observed (*r* = 0.322). The lowest strength was found with spirituality (*r* = 0.168). On the other hand, the associations with the ego climate showed a correlation with control under pressure (*p* < 0.01) being the positive and direct correlation and observing the greatest strength of correlation with this one (*r* = 0.195).

Considering the associations given with the levels of resilience, statistically significant differences are obtained with control under pressure (*p* < 0.01), adaptability (*p* < 0.01), optimism (*p* < 0.01) and spirituality (*p* < 0.05). All correlations are positive and direct, with the strongest correlation observed for control under pressure (*r* = 0.612), followed by optimism (*r* = 0.606). The lowest correlation strength occurred with spirituality (*r* = 0.167). In the case of control under pressure, statistically significant differences were observed with adaptability (*p* < 0.01), optimism (*p* < 0.01), spirituality (*p* < 0.01) and BMI (*p* < 0.05). All correlations are positive and direct, with the strongest correlation observed for optimism (*r* = 0.524). The lowest correlation strength was found with the BMI (*r* = 0.145). If the associations given with adaptability are taken into account, statistically significant differences are obtained with control and purpose (*p* < 0.01), spirituality (*p* < 0.01) and BMI (*p* < 0.05). All correlations are positive and direct, with the strongest correlation observed for control and purpose (*r* = 0.424), followed by spirituality (*r* = 0.413). The lowest correlation strength was found with the BMI (*r* = 0.159). In relation to the size of the control and purpose, statistically significant differences are obtained with the spirituality (*p* < 0.05), being the positive and direct relation and observing that the strength of correlation is (*r* = 0.162). Finally, for the associations given with the spiritual dimension, statistically significant differences were obtained with the BMI (*p* < 0.01), being the positive and direct relationship, observing the strength of correlation (*r* = 0.232).

## 4. Discussion

This research aims to define the motivational climate and its relationship with self-concept and resilience in a sample of primary schoolchildren in fifth and sixth grade primary education. In general terms, the literature shows similar studies carried out on university students and adolescents in the field of physical sports activity [17,47,48,49].

When analyzing the scores obtained in the motivational climate, it is verified that the schoolchildren obtain higher values in the task climate and its three dimensions, in comparison to the Climate Ego, data that coincide with the conclusion of investigations carried out by González-Cutre, Sicilia and Moreno [50], Almagro, Sáenz-López and Moreno-Murcia [20], Martínez, Cervello and Moreno [51]. This may be due to the fact that students orient their actions towards effort, persistence and personal improvement as well as valuing teamwork [52]. On the other hand, the averages referring to the ego climate and its dimensions were lower for the categories of UR and PE in González-Valero’s study [53]. With regard to the difference with gender, it can be observed how they show a greater tendency toward the Climate ego than they do [54,55]. Girls obtain higher values than boys in the dimensions of cooperative learning and important role; this can be explained by social factors because boys relate the practice of physical activity with a sporting and competitive factor in relation to women who are oriented toward leisure and recreation in a more cooperative context [31,54,55,56,57]. Following this line, the studies of Chacón or Cecchini, [49,58] showed higher parameters directed towards the ego climate in the male sex, a fact that attributes a different perception of abilities and competences according to gender.

As far as self-concept is concerned, it is shown that the general levels of self-concept are higher in males, as is the case for the academic, social and physical subcategories. This may be due to the fact that the male sex has a tendency to practice more hours of sport and this serves as a means of socialization and support in adverse situations as well as reinforcement between peers [59]. Physical self-concept is related to achievement goals associated with the ego climate and the competitive environment [60]. These results differ from those obtained in Molero’s study [61], where the female sex is mostly associated with the person’s physical self-concept, while the male sex is associated with strength and ego. This may be produced by gender differences, deducing the most critical view of women by their image, while men do not give it the same importance. It is also in the emotional and family dimension that girls have higher scores than boys. Salum-Fares [62] highlights the family dimension as the most important of all, since the family represents the basic social organization from which learning begins. This can be associated with the social, affective and contextual factors of the dimensions [63,64]. Reviewing the values obtained in the different dimensions of resilience, it could be observed that men had higher scores in all categories with the exception of control and purpose. This may be due to the fact that boys, being more related to sport, have greater resilience than girls. The practice of physical sports activity allows the subjects experience precise situations, where they will develop resilience with the passage of time [65]. However, these data are contrary to those established by Álvarez y Caceres [66] and Matalinares [67], who obtained significant differences in favor of women with respect to resilience capacity.

With regard to academic self-concept, schoolchildren have a high level of social and physical dimensions, which may be due to the fact that physical activity is associated with improved body composition and attractiveness [68]. Likewise, in relation to the academic dimension, children present a low homework climate, implying that schoolchildren or their parents prioritize their homework over the practice of physical activity. Authors such as Pinheiro [69] attribute to the family a pivotal influence for adolescents to practice sports and acquire healthy habits. The negative association between the persistence, emotional dimension and spirituality dimensions of resilience and academic self-concept has not been studied empirically as if it were a positive relationship [70,71]. This may be because by focusing on good performance, schoolchildren are not able to control pressure or resistance to discomfort. The same goes for spirituality, where they do not find the support of family, friends or teachers to overcome this pressure. These data confirm the idea of Masten [72], who identifies the links with certain figures (family network and teachers) as factors associated with a good resilient level.

In terms of social self-concept, schoolchildren show a high level of positive association with family status and body mass index. This can be explained by the family environment, since the family plays an important role in the emotional, social and intellectual development of the subject, with the family being the greatest social support available to participants. These results are close to those obtained by Caravedo [73] who determined that the self-concept levels of the children of separated parents are lower than the scores of the children of structured families. 

Considering the relationship of family self-concept and the dimension of optimism and adaptability to stressful situations, it can be established that respondents who show higher levels of PA have low scores in the control and purpose category. Works like those of Nearchou [74] suggest that having a high level of self-esteem can be a protective quality of negative results related to exposure to risk. These results can be explained by the low maturity of the participants, with the family being the factor that determines the behavior of the child at his or her earliest ages.

Another fact that is confirmed is that when the motivational climate associated with private recognition, based on self-learning, effort and individual improvement (task climate) increases, physical self-concept decreases. This can be due to the fact that the participants prioritize learning and cooperation, thus manifesting the search for the pleasure of practicing sports and diminishing the importance of the physical image they have. It happens in the same way when there is public recognition, which is based on social comparison (ego climate), the level of physical self-concept decreases. The relationship between physical self-concept and the ego climate is of less strength because in the ego dimension, one competes more and usually has a better physical condition and therefore a higher level of physical dimension [75].

It is also interesting to note that a greater capacity for resilience in respondents is associated with a decrease in physical self-concept, and no studies have been found to support these results. However, studies such as Garn’s [75] concluded that when the perception of personal competence (physical dimension) increases, the subjects’ ability to overcome negative experiences and overcome them is greater. It can be observed how the relationship between climate ego and task climate is negative and indirect—as one increases, the other decreases. These results may be due to the extent to which progress is made in the degree of specialization of sports practice and its mastery, progressively modifying the main motivation towards it. At the first level, sports practice focuses on promoting physical activity, cooperation and evaluation based on progress [76]. 

It is also important to highlight the direct relationship between task climate and levels of resilience, considering that the greater the intrinsic motivation based on learning and cooperation, the greater the values of resilience in children. Authors such as Hegberg and Tone [77] and Zurita [78] confirm this by recalling the benefits of practicing sports, through abilities such as optimism, control and purpose or emotional intelligence. As the results confirm, all the relationships between the different dimensions of resilience are positive and direct. This confirms that those research participants with a high level of resilience successfully overcome situations of stress, optimism, adversity and control in a much more remarkable way than those who obtain low scores in this capacity [79].

The present study reports a number of limitations. Amongst these, it should be highlighted that the descriptive and cross-sectional study design does not allow causal relations to be established. For these reasons, the results obtained in the present investigation should be interpreted with caution. The outcomes of the present study suggest that a practical implication could be to strengthen engagement with physical activity through physical education classes, as this is associated with better self-concept and resilience. This will lead to improved quality of life. In addition, it would be interesting to create motivational climates oriented towards the task, due to the greater benefits of these with respect to participation in playful physical activity.

## 5. Conclusions

Finally, it should be noted as the main conclusions of this study that men showed a greater tendency to climate ego, while women opt for task climate. As for the general self-concept, the average values were similar for both genders, with the academic, social and physical dimensions being emphasized in the male sex and the family dimension in the female sex. It has also been found that boys are more resilient than girls. 

It has been proven that the dimension of the physical self-concept is related to the motivational climate towards the physical activity, finding an indirect relation—when increasing the motivational climate towards the task, the physical dimension diminishes. Likewise, the relationship between resilience and the task climate is positive, finding that schoolchildren who tend to be motivated by cooperative work show greater resilience, while those with lower levels of resilience are more oriented towards the ego.

## Figures and Tables

**Table 1:** Motivational climate and self-concept levels according to gender.

Variable		Male	Female	Sig.
Motivational Climate	CL	3.99 ± 0.72	4.01 ± 0.76	*p* = 0.81
	EI	3.89 ± 0.47	3.82 ± 0.52	*p* = 0.28
	IR	4.11 ± 0.78	4.13 ± 0.74	*p* = 0.81
	PE	2.48 ± 0.78	2.26 ± 0.81	*p* = 0.04 *
	UR	2.50 ± 10.08	2.27 ± 10.04	*p* = 0.11
	MR	2.52 ± 10.01	2.24 ± 10.00	*p* = 0.05 *
	TC	4.00 ± 0.52	3.99 ± 0.54	*p* = 0.91
	EC	2.50 ± 0.80	2.36 ± 0.80	*p* = 0.32
Self-Concept	Academic	2.47 ± 0.48	2.30 ± 0.38	*p* = 0.01 **
	Social	2.91 ± 0.34	2.82 ± 0.35	*p* = 0.06
	Emotional	3.25 ± 0.59	3.25 ± 0.62	*p* = 0.98
	Family	2.91 ± 0.33	2.99 ± 0.23	*p* = 0.03 *
	Physical	3.91 ± 0.51	2.35 ± 0.53	*p* = 0.31
	General	2.78 ± 0.20	2.76 ± 0.21	*p* = 0.49

Note: TC, task climate; EC, ego climate; CL, cooperative learning; EI, effort/improvement; IR, important role; PE, punishment for errors; UR, unequal recognition; MR, member rivalry; *, statistically significant difference (*p* < 0.05); **, statistically significant difference (*p* < 0.01).

**Table 2:** Resilience levels according to gender.

	Dimensions	Male	Female	F	Sig
Resilience	Persistence	3.22 ± 0.59	3.15 ± 0.55	0.82	*p* = 0.36
	Control under pressure	2.95 ± 0.58	2.85 ± 0.62	10.32	*p* = 0.25
	Adaptability	2.95 ± 0.55	2.92 ± 0.57	0.12	*p* = 0.72
	Control and purpose	3.16 ± 0.68	3.17 ± 0.69	0.01	*p* = 0.89
	Spirituality	2.98 ± 0.76	2.93 ± 0.69	0.21	*p* = 0.64

**Table 3:** Correlation coefficients between motivational climate, self-concept levels, resilience and BMI.

	S	E	FAM	PHY	TC	EC	Pers	CUP	Adap	CAP	ESP	BMI
Academic	0.158 *	−0.106	0.025	0.249 **	−0.243 **	0.013	−0.176 *	−0.131	−0.317 **	−0.075	−0.302 **	−0.088
Social		0.107	0.151 *	0.049	−0.074	−0.037	−0.204 **	−0.136	0.018	−0.191 **	−0.042	0.155 *
Emotional			0.025	−0.113	0.010	−0.044	0.073	−0.072	0.089	0.026	0.011	0.050
Familiar				0.014	−0.045	0.006	−0.084	−0.044	−0.011	−0.226 **	0.058	0.078
Physical					−0.298 **	−0.149 *	−0.312 **	−0.282 **	−0.263 **	−0.268 **	−0.115	0.042
TC						−0.322 **	0.288 **	0.234 **	0.270 **	0.237 **	0.168 *	0.033
EC							−0.060	0.195 **	−0.070	−0.002	0.056	0.086
Pers								0.612 **	0.496 **	0.606 **	0.167 *	0.052
CUP									0.345 **	0.524 **	0.266 **	0.145 *
Adap										0.424**	0.413 **	0.159 *
CAP											0.162 *	0.111
ESP												0.232 **

Note: TC, task climate; EC, ego climate; S, social; E, emotional; FAM, familiar; PHY, physical; Pers, persistence; CUP, control under pressure; Adap, adaptability; CAP, control and purpose; ESP, Espirituality; BMI, body mass index. * Statistically significant differences at level (*p* < 0.05); ** statistically significant differences at level (*p* < 0.01).
